# Assessing contraceptive use as a continuum: outcomes of a qualitative assessment of the contraceptive journey

**DOI:** 10.1186/s12978-023-01573-4

**Published:** 2023-02-15

**Authors:** Rebecca G. Simmons, Jami Baayd, Megan Waters, Zoë Diener, David K. Turok, Jessica N. Sanders

**Affiliations:** Division of Family Planning, Department of Obstetrics & Gynecology, University of Utah, 30 North 1900 East, Salt Lake City, UT 84132 USA

**Keywords:** Contraception, Family planning, Contraceptive journey

## Abstract

**Background:**

Contraceptive use is often a multi-decade experience for people who can become pregnant, yet few studies have assessed how this ongoing process impacts contraceptive decision-making in the context of the reproductive life course.

**Methods:**

We conducted in-depth interviews assessing the contraceptive journeys of 33 reproductive-aged people who had previously received no-cost contraception through a contraceptive initiative in Utah. We coded these interviews using modified grounded theory.

**Results:**

A person’s contraceptive journey occurred in four phases: identification of need, method initiation, method use, and method discontinuation. Within these phases, there were five main areas of decisional influence: physiological factors, values, experiences, circumstances, and relationships. Participant stories demonstrated the ongoing and complex process of navigating contraception across these ever-changing aspects. Individuals stressed the lack of any “right” method of contraception in decision-making and advised healthcare providers to approach contraceptive conversations and provision from positions of method neutrality and whole-person perspectives.

**Conclusions:**

Contraception is a unique health intervention that requires ongoing decision-making without a particular “right” answer. As such, change over time is normal, more method options are needed, and contraceptive counseling should account for a person’s contraceptive journey.

## Background

The reproductive lifespan for people who can become pregnant can span approximately 35 years [1]. During that time, the average individual in the United States will spend approximately three to five of those years trying for, being, and recovering from pregnancy. This means, for many people, the vast majority of the reproductive lifespan is spent attempting to avoid pregnancy, relying on contraception to achieve that aim.

That the reproductive life course is long is obvious; yet, to date, theories assessing contraceptive decision-making tend only to account for short-term aspects of decision-making. Standard contraceptive indicators include unmet need, uptake, continuous use, switching, discontinuation, and satisfaction [2, 3]. These indicators approach contraceptive use specific to a particular method at a particular timepoint. Yet, when taken in the context of an entire reproductive life, such indicators fall short as they are influenced by where a person is in their reproductive life course, experiences with previous methods, and other decisional influencers not easily captured in standard variables.

Many research studies have placed high levels of contraceptive uptake and continuation as indicators of success and contraceptive switching and discontinuation (or discontinuation whilst still wanting to avoid pregnancy) as negative behaviors to be addressed through public health programs and policies [4–8]. Yet, when taken in the broader reproductive life context, all these behaviors are regular and expected over a 30-year life course. With more than 18 different methods, each with different attributes, side effects, costs, and availability, a person’s interest in and ability to use various methods will likely shift multiple times over their reproductive life.

A broader perspective on contraceptive decision-making which incorporates method use over the reproductive life course could provide insight into understudied areas of contraceptive use, such as how decisions are made to choose, start, use and discontinue methods, and the common influencers and factors in each of these decisions over time, or the “contraceptive journey.” The term “contraceptive journey” was coined by a participant in a previous study assessing contraceptive decision-making and is used here to denote the full experience of an individual making contraceptive choices over the course of their reproductive life [9, 10]. The purpose of this study builds on this idea and aims to develop a preliminary framework of contraceptive decision-making within the broader context of the full reproductive life course, to provide groundwork for research and policy advances and improve counseling. This project was conceived, in part, due to the individual contraceptive journey of the primary author who has used multiple methods (including long-acting methods and fertility awareness-based methods) over the course of her reproductive life and who has subsequently studied switching and discontinuation behaviors among HER Salt Lake participants [11]. This research is largely the result of not seeing method use conceptualized in a manner consistent with both personal and professional experience.

## Methods

The purpose of this research was to develop a theory of how contraceptive decision-making occurs over time. As such, we used a modified grounded theory approach for this project. We use the term, “modified” because we did not adhere strictly to a particular grounded theory approach (e.g., Straussian, constructivist), but rather utilized the broad concepts of grounded theory to pursue our line of inquiry. Our initial mode of inquiry was inductive, followed by abductive reasoning. Our analyses employed multiple grounded theory strategies (coding data, memoing, theoretical sampling, exploration of negative cases) to identify outcomes [12]. Our epistemological view of this research is pragmatic: we expected multiple perspectives and views of reality within the question of contraceptive experience and did not make assumptions of interviewer neutrality. Rather, our collective experiences as contraceptive users allowed us to engage with the variety of experiences we heard from participants in an informed way we did not view as separate, but rather as a facilitator to interpretation and meaning-making. Components of our methodology can be identified in the COREQ checklist.

Participants were individuals who had previously or were currently participating in the HER Salt Lake Contraceptive Initiative study—a county-wide contraceptive demonstration project providing no-cost contraception to people living in Utah [11]. Initially, we used the acronym HER to stand for “highly effective reversible methods (HERC);” however, as the initiative progressed, the initiative was simply referred to as HER Salt Lake, as the initiative focused on supporting all method choice. The purpose of the HER initiative was to assess long-term outcomes (e.g., pregnancy, educational attainment, sexual satisfaction) among people who received no-cost access to their preferred methods of contraception during the initiative. The HER initiative included anyone who could become pregnant who wished to avoid pregnancy for at least 1 year. Specifically, this included both participants who identified as women, as well as gender-expansive individuals. The decision to recruit from the HER Salt Lake population came, in part, because we felt we would get a wide and diverse participant group of known contraceptive users. For the parent study, individuals seeking new contraception at any of the four participating clinics were offered enrollment in a longitudinal arm of the study, which conducted surveys about contraceptive use and experiences for 36 months following enrollment. Participants were asked whether they were willing to be contacted for future studies. Among participants we identified three groups: those who continued their initial HER method, those who switched their method within the first 6 months of the study, and those who discontinued their method within the first 6 months of the study and were not using a subsequent method. As we wanted to ensure our study captured all experiences of contraceptive use (including decisional pathways around switching and discontinuation), we recruited individuals via email from each group in blocks of five until we reached thematic saturation.

The University of Utah Institutional Review Board approved this study (IRB #00102382). Individuals who agreed to participate were consented prior to the interview. Participants were invited to interview via telephone or in-person. All interviews were conducted by the lead author (RS) and a trained research assistant (MW). Both interviewers identify as female and had prior experience and training in conducting qualitative interviews. Prior to beginning the interview, each interviewer provided an overview of the purpose of the study and offered an opportunity for participants to ask any clarifying questions about the research and/or the interviewer’s background.

We developed a brief interview guide which asked participants to chronologize their contraceptive use, queried decisional aspects around initiation and cessation of each method used, and assessed how method experiences factored into subsequent decisions. The interview guide was pilot tested on 5 non-HER, reproductive-aged individuals and refined to reflect feedback and comments.

Participants who completed the interview received a $40 Amazon gift card. Interviews were audio recorded and then transcribed verbatim using a professional transcription service. Interviewers also took field notes during the interview calls which were incorporated as pre-memos within analyses. The two interviewers held biweekly meetings during the interviewing process reviewing field notes and discussing interview experiences, until both interviewers agreed that the interviews were not identifying substantively new thematic constructs. Saturation was assessed both during the interview process and again during the coding process.

For our analyses, the lead author (RS) and two trained research assistants (MW and ZD) conducted open coding on five of the interviews collectively, developing initial codes and memo-ing together, to get a broad sense of the data as well as begin to define codes. Initial codes were broad, such as “side effects” as a code to capture any notation of side effects, from increased bleeding to mental health. We conducted double-open coding for each interview, both adding to the codebook as necessary and eliminating redundant codes. This initial open coding was followed by axial coding, to identify groupings of codes (e.g., overlap between beliefs around contraceptive responsibility and partner triggers for seeking contraception). We then conducted selective analyses, assessing substantive significance of findings across axial nodes to develop a contraceptive journey framework [13]. Transcripts were coded using the qualitative data analysis software Dedoose [14].

## Results

A total of 190 HER participants were contacted via email. Among those, 3 people declined to participate, 2 participants had email addresses that no longer worked, and 152 people did not respond to the email request within the 2-week response window. Ultimately, 33 people provided interviews between July 1, 2018 and August 31, 2018. All interviews were conducted telephonically with only the researcher and the participant present. While our study initially aimed for around 30 participants or until saturation was reached, after coding some initial interviews, we made the decision to specifically recruit more individuals who reported experiences of pregnancy and parenting in the HER Salt Lake surveys, as we felt contraceptive journeys were likely influenced by these life events. The average interview took 45 min to complete. Socio-demographic information about participants is provided in Table 1.

**Table 1:** Sociodemographic characteristics of participants in the HER Contraceptive Journey study

Sociodemographic characteristics	n (%)
Age at HER contraceptive journey enrollment	
18–19	7 (21.2%)
20–24	17 (51.5%)
25–29	7 (21.2%)
30–34	2 (6.1%)
Race/ethnicity	
American Indian/Alaska Native	1 (3.0%)
Black	1 (3.0%)
Hispanic/Latina	6 (18.2%)
White	21 (63.6%)
Other	4 (12.1%)
Educational attainment	
High school/GED	7 (21.2%)
Vocational/technical training	1 (3.0%)
Associates/some college	15 (45.4%)
4-year degree	6 (18.2%)
Graduate/professional education	2 (6.1%)
Prefer not to answer	2 (6.1%)
Sexual identity	
Bisexual	2 (6.3%)
Exclusively heterosexual	25 (78.1%)
Mostly gay/lesbian	1 (3.1%)
Mostly heterosexual	4 (12.5%)
Ever been pregnant	
Yes	6 (18.7%)
No	26 (81.3%)
Religion^a^	
Christian (Protestant, Evangelical, etc.)	4 (28.6%)
Mormon (The Church of Jesus Christ of Latter-Day Saints)	3 (21.4%)
Not religious	5 (35.7%)
Other	1 (7.1%)
Don’t know/prefer not to answer	1 (7.1%)
Relationship status at HER enrollment	
Actively dating but not in a committed relationship	7 (22.6%)
Living together, but not married	16 (51.6%)
Married	1 (3.2%)
Single, not in a relationship	6 (19.4%)
Prefer not to answer	1 (3.2%)
Initial contraceptive method accessed through HER Salt Lake	
Combined oral contraceptive	9 (27.3%)
Copper intrauterine device (IUD)	6 (18.2%)
Hormonal IUD	9 (27.3%)
Implant	4 (12.1%)
Injection	4 (12.1%)
Vaginal ring	1 (3.0%)

^a^Religion was asked in a subsequent follow-up survey and thus, not all participant responses were captured

A person’s contraceptive journey occurred in four *phases*: (a) identification of the need for contraception (either to obtain a method or a decision to begin use); (b) the process of initiation/method selection; (c) experiences with a method during use; (d) cessation and decisions to stop use. Each phase of the process is the result of a series of decisions and experiences that were often discussed separately by participants. For example, a person’s decision to begin using contraception is distinct from the experience they had with selecting and initiating a particular method (e.g., the conversation with a provider, buying it from a store or pharmacy, having it inserted). These prior phases may play into their experiences with the method during ongoing use (for example, a painful/negative IUD insertion could result in ongoing negative feelings about the IUD), but they are often conceptualized as being separate components of each story.

Across all phases of the contraceptive journey, we identified five *factors* that most heavily influenced participant decision-making: physiological factors, values, experiences, circumstances, and relationships. Examples of these factors within each contraceptive phase are demonstrated in Table 2. Each phase of a contraceptive journey may contain one or more elements of these decisional components.

**Table 2:** Contraceptive phases and factors associated with contraceptive decision-making as part of the contraceptive journey

Contraceptive phases	Factors influencing contraceptive decision-making				
	Physiological factors How do pre-existing health conditions, experiences of side effects, adverse events, and other physiological impacts of contraception influence subsequent decision-making?	Values How do personal considerations such as religious beliefs, beliefs about sex and relationships, cultural influences, and pregnancy desires impact method decisions?	Relationships How do sex partners, family members, friends, health care providers and other individuals in a person’s life influence their contraceptive decision-making?	Circumstances How do externalities such as geography, income level, employment status, insurance status and other circumstantial factors exert themselves in a contraceptive choice?	Experiences How do life experiences, including those with previous contraceptive methods, the healthcare system, birth/abortion, sexual violence/coercion play a role in method decisions?
Identification of need How does someone get to the place where they decide they need a new method?	*I had really bad cramps and really heavy periods. So, I got on it [contraception] mostly for that. Also with the hope that it would get rid of my acne, but it did not* *–Amaia, age 27*	*It was like, a year and a half before I started any other contraceptive, just because I was so focused on being a mom. I didn’t have any kind of interest in pursuing relationships or even having sex. So, I just didn’t see the point of having any kind of birth control. I wanted my body to heal from my pregnancy and get back in shape and take care of myself mentally because of the whole new transition into being a new mom and everything* *—Kiara, age 26*	*“As a young teenage girl, I was excited to start the pill because I had seen and heard some of my friends who are on the pill, who their boobs got bigger, maybe their butt got bigger. You know, I was all about that.”* *—Valerie, age 34*	*At the time, I just was really sure that I needed something foolproof because at that point, I was really traveling a lot and it was hard. I wasn’t seeing my regular doctor anymore and every time I moved, if I did have to go to the doctor, I was usually going to urgent care or something and it was difficult to do certain things remotely…I was like, I don’t know, I may go to Asia, I may go backpacking or something after this and I don’t want to always be worried if I can’t get in touch with my doctor. —Romina, age 29*	*That was also a lot of the reason why I kind of always wanted to be on contraception, because when there were incidences where I felt like I wasn’t safe or I couldn’t get out of a situation, I always wanted to be on contraception so that way I didn’t have to worry about rape and pregnancy.”* *—Begonia, age 26*
Initiation What is the initial experience with the method? What is the process of getting a new method?	*Ok, so the first time I used… I had sex I pretty much did not know that I was allergic to condoms. So now I know I’m allergic to condoms. Latex, that’s what it is* *—Mari, age 25*	*I don’t think it’s [hormonal contraception] is for every body and all the different types I’ve tried have told me that. Now I’m reading a really great book about fertility in women and your body and I’m trying to get better at the fertility awareness method. I’m just trying to be more in tune with my body instead of just ignoring it and flatlining everything* *—Roxanna, age 31*	*“I always wanted to use condoms. So, I always used condoms. And then one boy asked, ‘Are you on birth control?’ And I said…at the time, I liked and said yes because I didn’t want him to freak out and then the next day I went and got Plan B because we hadn’t used protection.” —Elle, age 24*	*When I got the Skyla, my doctor put it through my insurance, and she thought it was covered. I came home and discovered I had a bill for $300 and I was supposed to be on a payment plan. And I was supposed to petition them or something and forgot. —Romina, age 29*	*I felt comfortable with telling them I had started a new relationship and wanted a new method. And I explained the ones I had already tried and why they didn’t work. I think they slightly were pressuring me to do the pill. But I was like, “Alright, I don’t think you know how much I would forget to take the pill, so that’s a bad method for me.” Elle, age 24*
Continuation What are the ongoing experiences of using a contraceptive method? What happens after uptake?	*And my struggle with the IUD was that I remember looking up and I was just doing research and seeing, they said you typically have really bad cramps for six months or so… And I had really bad cramps for over a year…And it was awful. But I don’t know, just eventually it went away… I almost got it taken out a few times, but they kept telling me it was normal. And so I stuck it out. Now that I have none of the side effects, it’s fantastic* ***—****Syndey, age 25*	*“I just did not take it consistently. I feel like I had some weird religious guilt during the time, so I think I would go through phases of being like, ‘Oh, I don’t want to have sex’ and then wanting to have sex and having sex. And so I think that’s why the pill didn’t really stick with me then, because I would go through phases of being like, ‘I don’t really need this.’ And that’s not really how you take the pill and it works.” —Selena, age 26*	“*Initially I liked it [vaginal ring]. But I just had really bad depression on it and it was my freshman year in college and I was just so depressed on it and it really decreased my libido. So I was having all these issues with it, so my stepmom was just like, “it takes your body a little while to acclimate, so just try to keep it in.”* *—Selena, age 26*	*The pill was the easiest for me to get and it was the cheapest. But what I didn’t like about it was that I often forgot to take it and forgetting would make me super anxious about getting pregnant afterwards. So I really wanted an IUD because then I wouldn’t have to think about it or worry about it for years. And so I was looking into that, but I saw the prices. It was over my budget. I remember it was super expensive. So I stuck with the pills because I wasn’t able to afford that* *—Sydney, age 25*	*And then, when I started the pill, got married and had been on it for probably seven or eight months…I was taking a pathophysiology class and the professor told us a story of a girl who had just gotten married and was on the pill and had a stroke because she got a blood clot. And all of a sudden, I started getting super paranoid and nervous because I remembered I had a cousin who had, like, a blood clot when she was pregnant on an airplane. And so, I like couldn’t stop thinking about it, decided to go get my blood tested for it and it turns out I did have it…so then the doctor was like, “Okay you should not be on this pill because there’s estrogen in it and the chances of a blood clot were really high.” —Alexis, age 28*
Cessation What leads someone to decide to stop using a method?	*I had to go inpatient for suicidal ideation. I think it was at that point where they were like, “We want to limit the amount of pills you’re on so that we can figure out what you’re [experiencing].” They kind of wanted to mainline me so that I wasn’t having as many mood swings and stuff. So I think it was at that point when I stopped taking the birth control pill. —Lydia, age 29*	*I have tried basically every hormonal type, except for the Mirena IUD and realized all those times that for me and my mental health I needed to look for something different. I don’t want to feel like I’m foreign within myself and that’s honestly how I felt when I was on all those other birth controls. I felt more like a stranger. —Kiara, age 26*	*“My parents had heard some information that the patch caused, like, blood clots and some other harmful things so they wanted me to stop taking the patch and start taking the pill.” —Valerie, age 34*	*Well, I didn’t have insurance and just paying out-of-pocket, it was getting really expensive. That was a big reason [to stop birth control pills] as well. —Romina, age 29*	*I was using them [condoms] pretty religiously in the sense that I wouldn’t have sex without them. But somehow, I don’t know, but it must have broken or something. But I did end up getting pregnant in high school and just the whole decision of having to decide to get an abortion and figuring out the right path for me…just afterwards I didn’t want anything to do with them ever again. —Izzy, age 24*

Physiological factors refer to elements of contraception decision-making associated with health or health outcomes. Applied to the phases of the contraceptive journey, this could mean that a person may have limited choices in the *decision-making* phase due to contraindications, such as migraine with aura or a family history of breast cancer. In the *initiation phase*, physiological factors could be at play with, for example, a difficult IUD placement due to a bicornate uterus. During *continuation*, physiologic factors include experiences of side effects, such as increased bleeding or vaginal dryness during sexual intercourse, which often resulted in participants feeling disappointed, stressed, or as though something was wrong with them as individuals. Physiologic factors leading to *cessation* could include a certain tipping point wherein a user cannot tolerate side effects any longer, or an unexpected adverse event, such as a deep vein thrombosis.

Values include how personal beliefs, religious beliefs, cultural influencers and pregnancy desires factor into the contraceptive phases. In the *decision-making* phase, for example, an individual could rule out consideration of any hormone-based methods, due to religious reasons. During *method initiation*, values may influence how willing a person is to pursue methods that are more difficult to access, such driving a long distance to find clinic that will provide an IUD as emergency contraception in order to have greater assurance of not becoming pregnant. Cultural or religious values around sex and sexuality may influence a person’s *continuation* of methods, if shame factors into their use. Participants reported *cessation* of hormonal methods in order to feel like themselves again, or to feel aligned with their bodies.

The relationship construct includes relationships with intimate partners and health care providers, but also with friends, parents and other family members. Many participants reported interest in *initiating* a method after hearing a positive story from a friend or family member. Other participants reported that their parents or others played a supportive role in helping them start a method, by taking them to their doctor visits, or helping to pay for more costly methods. Support for *continuation* could look like a partner reminding the person to take their methods consistently, or willingness to use condoms at every sex act. Many participants experiencing challenges with a method felt empowered to *stop using it*, after sharing their stories with friends or family members and realizing that they weren’t alone in experiencing those challenges.

The circumstances factor incorporates all external components that facilitate or hinder method use, including things like employment, geography, insurance status, access to transportation, and other elements, such as age. For example, participants commonly made decisions about which *method to initiate* based on which methods they could afford or their insurance would cover. Similarly, some participants reported gatekeeping by pharmacists or providers for methods like emergency contraception or sterilization, due to being considered “too young.” Participants working irregular jobs or hours often had trouble *continuing* using certain methods, due to the difficulty in getting to a pharmacist or a doctor regularly. Many people reported *cessation* of a particular method due to moving locations, changing jobs/insurance, or other life changes.

The experiences construct includes past experiences with contraception, but also, more globally, life experiences. For example, a person who became pregnant despite using a condom may choose to never *initiate* condom use in the future. Experiences of sexual violence may result in an increased hesitancy or challenge in inserting IUDs. *Continuation* of a particular method could be a challenge, if the participant had previously experienced negative side effects from prior use and was therefore “expecting” the method to backfire at any moment. Participants who had used multiple methods often reported an increased willingness to *cease* subsequent methods faster, if they had negative experiences, due to more self-knowledge and more understanding of what compromises they were willing to make.

**Fig. 1:**
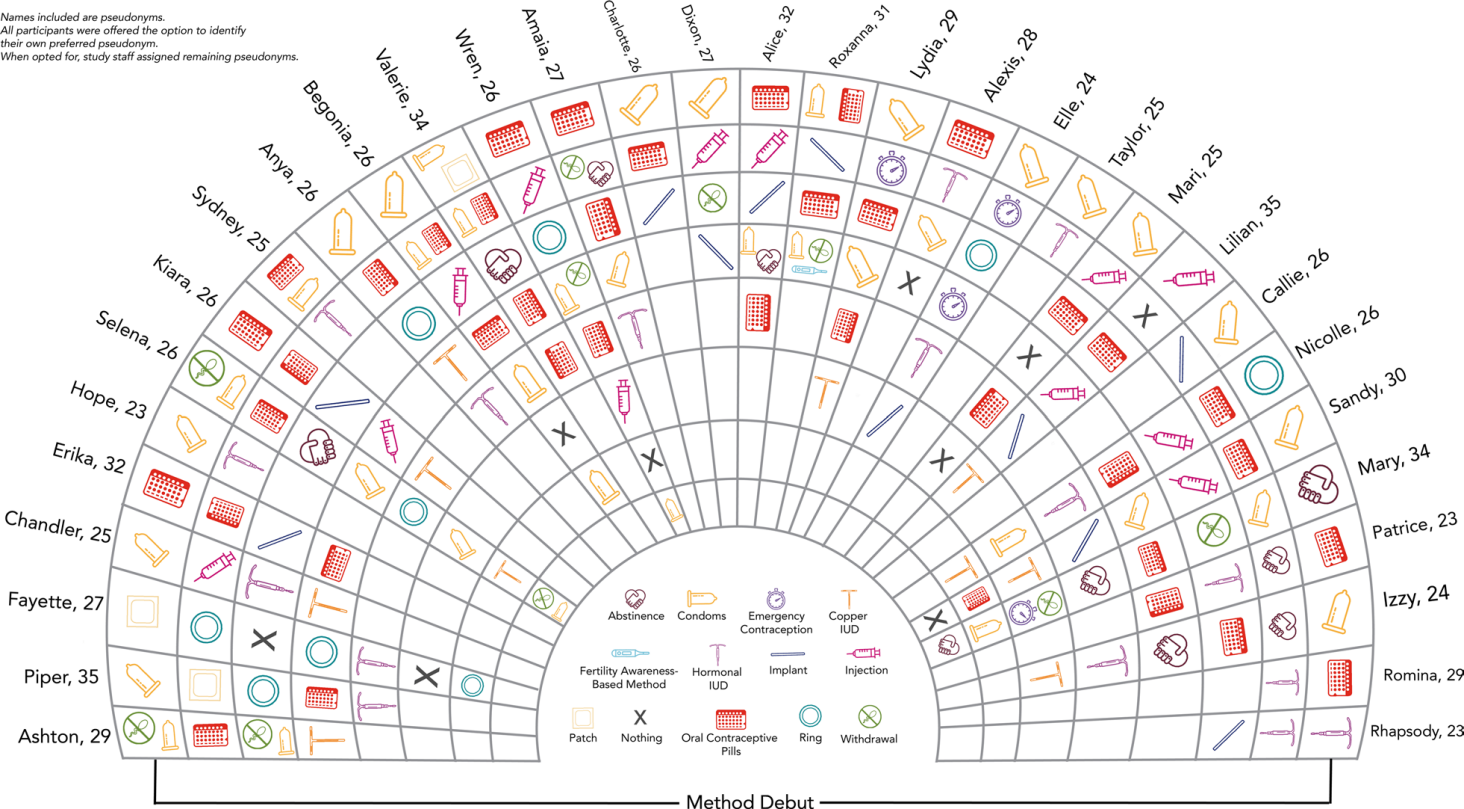
Individual contraceptive journeys of study participants. This graphic provides information about the contraceptive journeys of participants in the HER contraceptive journey study. Initial methods are shown on the outside circle, with subsequent methods represented in each following ring. One participant (Selena, age 26) reported 7 additional methods used which did not fit into the existing graphic: combined oral contraceptives; condoms and withdrawal; progestin-only pills; the copper intrauterine device; progestin-only pills; condoms, withdrawal and emergency contraception; combined oral contraceptives

Some participant stories are represented multiple times in Table 2. Most participants had journeys that crossed the *phases* and *factors* many times over their reproductive life course. Although most participants were early in their reproductive life (ages 18–24) during the initial interviews, most had already tried multiple methods, as shown in Fig. 1. Many participants described the journey as a component of self-discovery—navigating through the complexity of their bodies and lives to understand their needs in a given moment. Participants method choice was not linear—many returned to using previously used methods multiple times in their journeys. While many participants reported that efficacy was a strong consideration for some of their decisions, ultimately, journeys did not largely reflect a movement toward increasingly effective methods. Ultimately, prior contraceptive use fed into subsequent contraceptive decision-making and, rather than acting as single decision-points, represented a continuous process of assessment and response.

## Discussion

Findings from our study underscore the ongoing nature of contraceptive decision-making. For our participants, pregnancy avoidance was a part of their everyday lives for decades, and their decision-making around contraception was nuanced, multifactorial, and accounted for all of their previous experiences with contraception. Viewing contraception through this lens underscores several important constructs that participants identified that have also been demonstrated in other research, including the normalcy of contraceptive switching and discontinuation [15], the critical importance of comprehensive method availability [16, 17], the common experiences of using multiple concurrent methods [18], the decisional importance of past method use on future method decisions [10], and the neutrality of individual methods as positive or negative choices [19, 20].

We found that contraceptive decision-making occurs in a continuous manner with four distinct decisional points, which occur in an ongoing manner throughout the reproductive life course. These decision points interact with the five major realms of decisional influence. Many of the realms of decisional influence have overlap with existing frameworks of family planning use, such as the AAAQ framework [21] or the Family Planning Quality of Care Framework [22]. For example, components of accessibility, such as geographic accessibility and financial accessibility, fall within our model’s definition of “circumstances.” The construct of acceptability has elements of our framework’s categorization of “values,” and similarly, a key component of quality of care is provider-patient interactions, which fall under “relationships” in our model. Key differences in our framework compared to other existing frameworks is that the proposed model is not limited to actionable levers. For example, if an organization seeks to improve contraceptive access, use of the AAAQ framework or Bertrand’s accessibility framework [23] can identify activities that can reduce access barriers, but our findings also give space to existing elements within the contraceptive journey that are relatively immutable to external forces. For instance, it is wholly within reason to expect that making IUDs available as same-day emergency contraception within all clinics, for a reasonable price, provided by trained, culturally sensitive providers, will result in increased utilization of this approach to emergency contraception. Yet, a substantial proportion of people needing emergency contraception will still not select this approach. Our framework notes that other influences, such as a prior experience with IUDs [10], stories a person has heard from friends or family members (or other relational influencers), life circumstances (e.g., prioritizing getting back to work vs. taking additional time for an IUD insertion), or physiological factors (a history of yeast infections, for example) also weigh heavily into this decision and are individual, specific, mutually occurring, and exert decisional weight that is largely outside the realm of realistic public health influence. This is consistent with emerging conversations around the importance of honoring the concept of demand-side unmet need: the understanding that many women simply do not want to use contraception at a particular moment and attempts to change this are misguided [24].

As individuals move through their reproductive lives, changes to their relationships, values, circumstances, and bodies are inevitable and thus, ongoing changes to contraceptive strategies are expected and should be supported. This fluidity has been noted in other studies of contraceptive use [17]; our framework attempts to demonstrate why this fluidity occurs: contraceptive use is complex. For most preventive care services, the intent is to completely avoid a disease outcome. Yet, unlike other health conditions addressed preventive medicine, such as depression or sexually transmitted infections, pregnancy is not a disease outcome, but rather a complex life experience with multifactorial influences [25]. As such, using traditional medical models which prioritize effectiveness for disease prevention are often at odds with how contraception is viewed and used in real life. With more than 18 different method types and hundreds of variations of formularies, there have never been more contraceptive options than there are today. Yet, our study underscores the critical importance of both comprehensive method availability within health systems and the ongoing importance of prioritizing new method development to continue to create method options that respond to the diversity of end-user needs. Method options are critical to supporting people as they move through three or more decades of reproductive capacity. Many individuals in our study re-engaged with or continued to use methods that didn’t work for them, simply because they felt they had no other choice, but tolerance does not equal satisfaction. Ongoing use of methods which cause physical or mental distress, are unaligned with values, or do not meet relational or circumstantial needs is a lived reality for many individuals and seen as a price to pay in order to avoid pregnancy [26]. Yet this is a reality that is inconsistent with a human-rights framework of reproductive autonomy [27].

This study was conducted among a relatively young, homogeneous population of individuals who were mostly at the beginning of their contraceptive journeys. Yet, prior research on contraceptive journeys among more diverse populations has also identified some of the lived complexities found within our study [9, 10, 27–31]. Future studies assessing contraceptive journeys among individuals closer to the end of their reproductive lives, or who are in immediate post-abortion and post-partum periods may identify other areas of importance within a contraceptive journey framework. Our study also assesses individuals who received no-cost contraception through the parent study. While this aspect makes our study population different from the broader population, it does underscore the singularity of short-term contraceptive initiatives in the larger context of the contraceptive journey; for many participants, their exposure to the HER study was simply a single (though sometimes very important) event in a much larger process.

Viewing contraceptive use as an ongoing, ever-changing process offers new directions for research, as well as additional support for clinical equipoise in counseling and care provision. Other fields, including sociology and economics, have utilized the concept of “path dependence” theory, which posits that past events and decisions constrain future decisions [32–34]. Our findings support inclusion of these concepts in contraceptive research as well. The idea of a contraceptive journey underscores the importance of new method development, to ensure people have sufficient choices to account for their multi-factorial needs. It highlights the need for more assessment of contraceptive switching and discontinuation that is specifically based on how to improve these processes for users. For example, our findings support use of contraception decision support tools, such as the PICCK PHI tool, which incorporates a person’s contraceptive journey into method decision-making and conversations with providers [REF]. Including contraceptive journeys into contraceptive contextualization provides theoretical underpinnings that can potentially improve measurement around contraceptive decision-making. Possibly most importantly, it puts the stories of contraceptive use as a component that clinicians should care about when providing contraceptive care.

## Conclusions

There is no magic method that fits every person’s needs. There is no singular experience of contraceptive decision-making. Thus, nuance, method neutrality, and ongoing family planning provision, research, funding and strategic prioritization are likely the best ways to account for people’s lived realities throughout their reproductive lives.

## Data Availability

The datasets used and/or analyzed during the current study are available from the corresponding author on reasonable request.
